# An Examination of the Apparatus Proposed for the Quantitative Administration of Chloroform

**Published:** 1904-07-30

**Authors:** 


### AN EXAMINATION OF THE APPARATUS PROPOSED FOR THE QUANTITATIVE ADMINISTRATION OF CHLOROFORM.

Dr. Waller and Mr. Wells [1] have conducted an inquiry into the efficiency of three forms of apparatus designed for graduated administration of chloroform for anæsthetic purposes. First, the Dubois pump, which, it is claimed, delivers continuously, at any convenient rate between 4 and 20 litres per minute, air charged with chloroform vapour at the nominal percentage of 1·2, 1·6, and 2, was found to work easily and well, and the chloroform percentage remained below 2 while the pump was working. A loose face piece should be used and the anæsthetic mixture supplied freely. The drawbacks are the cost (£20), and the fact that the instrument is rather complicated and heavy. The surplus chloroform may be inconvenient to anæsthetist and operator but escaping as it does into the air of the theatre, the inconvenience, owing to the considerable dilution, is trifling.

The Junker apparatus (Krohne and Sesemann) consists of a face piece, freely open to the air, from which the patient inspires definite volumes of chloroform by means of a small accessory pump that drives measured amounts of air through a bottle containing liquid chloroform. The idea is to deliver a just sufficient proportion of chloroform vapour at the commencement of inspiration, which is practically a very difficult matter to realise, especially as the volume of inspiration varies. The tax on the attention of the administrator is very heavy, and he is liable frequently to fail in securing the exact synchronism between inspiration and chloroform delivery which is essential to complete success with this form of apparatus.

The Harcourt inhaler (Messrs. Griffin and Co) consists of a bottle, tap, and tightly-fitting face piece. The patient's inspirations draw air through chloroform in the bottle. This is theoretically an unsound principle, because the mask must fit the face perfectly; the bottle must not be shaken in the least; the patient must be motionless, and the respirations absolutely even if the nominal percentage of chloroform is to be uniformly respired. A single shake of the bottle momentarily raises the percentage to more than double its nominal value. With weakening respiration and slower air draft through the chloroform bottle, the percentage of chloroform inspired is also raised. The instrument is portable and cheap. As a result of his researches Dr. Waller makes the following statements:—

(1) Chloroform is not an uncertain agent. The effects it produces are proportional to the mass in which it acts. (2) For ordinary anæsthesia of the human subject the chloroform inspired should be between the limits of 1 and 2 per cent. (3) Death by chloroform is, in the great majority of cases, death by simple overdose. (4) In the three forms of apparatus professing to deliver graduated amounts of chloroform, the delivery under laboratory conditions is practically what it professes to be. The choice of apparatus is a matter of clinical convenience.

Mr. Legge Symes[2] investigated the concentration of chloroform vapour in air drawn from beneath a Skinner's mask. Chloroform was applied to the mask by continuous dropping or a single douche, and the air aspirated by means of bellows and sampled in a densimeter bulb.

With moderate and approximately equal quantities of chloroform under ordinary conditions, the mask yields vapour between the desirable limits, but reaching as high as 3 or 4 per cent. when chloroform is copiously applied.

The more open the fabric of which the mask is made, the higher the concentration of the vapour. By the continuous drop method the desired concentration is more uniformly sustained. Changes in respiration produce variation in concentration, and fall in frequency and depth of respiration may lead to inhalation of dangerously concentrated vapour, even when the amount of chloroform is apparently not excessive.

[1] Lancet, July 9, 1904. [2] Ibid.

